# Bioactivity Profiling of Chemically Characterized Extract of Saudi Jackfruit (*Artocarpus heterophyllus*) Using In Vitro and In Silico Approaches

**DOI:** 10.1155/sci5/8015648

**Published:** 2025-06-22

**Authors:** Hanan Y. Aati, Renad Al-Arifi, Chitchamai Ovatlarnporn, Abdul Rauf, Abdul Basit, Huma Rao, Maria Batool, Kashif ur Rehman Khan

**Affiliations:** ^1^Department of Pharmacognosy, College of Pharmacy, King Saud University, P.O. Box 2457, Riyadh 11451, Saudi Arabia; ^2^Department of Pharmaceutical Chemistry, Faculty of Pharmaceutical Sciences, Prince of Songkla University, Hat Yai 90110, Songkhla, Thailand; ^3^Drug Delivery System Excellence Center, Faculty of Pharmaceutical Sciences, Prince of Songkla University, Hat Yai 90110, Songkhla, Thailand; ^4^Department of Medicinal Chemistry, College of Pharmacy, Shenzhen Technology University, Shenzhen 518118, Guangdong, China; ^5^Department of Pharmaceutical Chemistry, Faculty of Pharmacy, The Islamia University of Bahawalpur, Bahawalpur 63100, Punjab, Pakistan

**Keywords:** antibacterial, anticancer, antioxidant, *Artocarpus heterophyllus*, chemical profiling, computational studies

## Abstract

*Artocarpus heterophyllus*, also known as jackfruit, is recently introduced for cultivation in Saudi Arabia. In this study, the ethanolic extract of *Artocarpus heterophyllus* fruits (EAHF) was explored for its chemical and pharmacological properties. The EAHF was revealed with total phenolic contents (115.5 ± 5 mg GA.Eq.gm^−1^ DE), total flavonoid contents (77 ± 2.22 mg QU.Eq.gm^−1^ DE), and total tannin contents (59.33 ± 1.66 mg TA.Eq.gm^−1^ DE), while 81 phytocompounds were identified by the GC–MS analysis. The extract was found with maximum antioxidant activity in the FRAP method. EAHF showed significant thrombolytic (85.51%) and hemolytic (1.01%) activities. EAHF showed strong enzyme inhibition, against urease (95.65%), tyrosinase(71.01%), and α-amylase(28.17%). The extract showed cytotoxicity potential against breast cancer cell lines (MCF-7 and MDA-MB-231) in a concentration and time-dependent manner. Furthermore, selected compounds from the GC–MS profile were tested for in silico toxicity, ADMET, and molecular docking study to analyze the interactions between compounds and selected enzymes. Overall, the findings of the study suggest that the EAHF has pharmacological potential and could be a suitable therapeutic alternative for various common diseases. However, further, in vivo toxicological and pharmacological investigations are recommended on this extract.

## 1. Introduction

The research on therapeuticaly relevant food plants provides compelling evidence that plant based natural products with antioxidant activity can shield biological systems from oxidative stress [1]. Reactive oxygen species (ROS) are highly reactive molecules derived from oxygen. ROS can seriously harm cells and tissues during infections and several degenerative conditions, including aging, cancer, diabetes, and neurodegenerative illnesses, i.e., Alzheimer's disease [2]. Diabetes is a chronic metabolic disease characterized by abnormally high blood glucose levels due to insufficient insulin secretion by pancreatic cells and/or resistance to the antidiabetic hormone, insulin. When the oxidative step in the glycation process occurs, the process is referred to as glycoxidation. Advanced glycation end products (AGEs) are compounds that are produced during glycoxidation, along with free radicals and byproducts of the autooxidation of the glycating sugar. Radical scavengers and antioxidants block these processes. In this regard, several natural extracts, including polyphenol-rich extracts have shown notable effects. It is well known that rutin, curcumin, and garcinol extracts have antioxidant qualities and prevent the production of AGEs. Furthermore, recent studies suggest that substances with combined antioxidant and antiglycation properties work better to treat diabetes mellitus.

Similarly, the neurodegenerative diseases are thought to have a close correlation with the oxidative stress. Alzheimer's disease is reported with increasing progression mediated by elevated levels of free radical species in the body. In this way, the functional foods that have antioxidant potentials are seen with attenuated potential against the free radical–mediated disruption in the pathophysiology of Alzheimer's disease. It is noteworthy to mention the effects of oxidative stress on the skin. Almost every skin problem originates from the oxidants in one or the other stage. Melanin is an important pigment that avoids the damage of the skin. Melanin levels in the body are greatly affected by various factors including tyrosinase enzyme production. The higher release of tyrosinase along with elevated levels of free radicals can cause the depletion of melanin pigment that in turn could lead to various skin diseases including cancer. Therefore, the use of agents that have free radical scavenging properties as well as tyrosinase inhibition properties has proved vital to treat and avoid skin pathophysiological alterations.

Despite biomedical advancements, the progression and increasing prevalence of cancer remain to continue. Breast cancer is the second most prevalent cancer after lung cancer. The currently available treatment options for breast cancer are associated with severe side effects that limit the compliance rate of the therapies. The pathophysiology of breast cancer has recently been explored in terms of its link with ROS levels in the species. It has been reported in various investigations that breast cancer progression is highly mediated by oxidative stress. The use of vegetables and fruits which possess antiradical properties is seen with preventive effects on breast cancer. In the above context, the inclusion of vegetables and fruits with antioxidant potential is very important to diminish the free radicals and maintain the normal physiology of the body.

The various phytoconstituents in medicinal food plants are reported with key contributory roles behind the pharmacological effects of the medicinal food plants. Jackfruit is through to exert antidiabetic and other therapeutic effects through ascorbic acid which is the most abundant phytochemical in jackfruit, with beta-carotene and lycopene following closely behind. Numerous studies revealed that diabetic patients had lower basal vitamin C levels, and there is also evidence to suggest that diabetes is associated with an increase in oxidative stress. Eating foods high in ascorbic acid can decrease the risk of developing diabetes [3].

The hemolytic activity of a compound serves as an indicator of its overall cytotoxicity to healthy, normal cells. Saponins, a class of phytochemicals derived from plants, are known to exhibit hemolytic activity through the modification of red blood cell membranes. The spectroscopic method employed in in vitro hemolytic assays allows for the straightforward and effective quantitative measurement of hemolysis. This technique facilitates the evaluation of the effects of varying concentrations of biomolecules on human erythrocytes [4]. Thrombus formation in blood vessels can lead to serious complications such as atherothrombotic diseases, i.e., cerebral or myocardial infarction. To break up clots that have already formed in the blood vessels, thrombolytic agents, such as urokinase, tissue plasminogen activator, and streptokinase (SK), are used. However, these medications have some drawbacks that can result in severe anaphylactic reactions, bleeding, lack of specificity, and other dangerous and occasionally fatal outcomes. Furthermore, a patient may not receive more than one SK treatment due to immunogenicity. Plant-based agents should be less antigenic and more affordable [5].

*Artocarpus heterophyllus* also known as jackfruit belongs to the family Moraceae. The plant stays green throughout the year and can grow between 8 and 25 m and have a stem diameter of 30–80 cm (12–32 inches). The top of the tree is usually in the shape of a cone [6]. There have been traditional uses for the Artocarpus species in the treatment of different diseases as medications. The various plants of the genus have been utilized for their antimicrobial, antidiabetic, antioxidant, anti-inflammatory, and anthelminthic properties. The phytonutrients found in seeds, such as lignans, saponins, and isoflavones, are beneficial to human health. The high fiber content of jackfruit facilitates easy bowel movements and enhances the digestive system. It is mostly cultivated in tropical countries such as Burma, Bangladesh, India, Brazil, Thailand, Indonesia, Philippines, Sri Lanka, Pakistan, and Malaysia [7]. Recently, jackfruit has been included in the flora of Saudi cultivation for sustainable food sources with therapeutic and preventive potential against several medical conditions. The various cultivars of a plant species may have various phytochemical and biological properties. It is because soil composition varies among different regions of the world that hugely impacts the chemical composition of plant species either positively or negatively and that ultimately results in the change of pharmacological and toxicological profiles of the species [8, 9]. Therefore, it is of great need to evaluate the phytochemical and pharmacological profiles of various cultivars of plant species. Therefore, in this research, an in-depth multifaceted approach was followed to explore the chemical profile of Saudi cultivar *A. heterophyllus* using quantitative polyphenolic estimation assays and gas chromatography–mass spectrometry (GC–MS) analysis as well as pharmacological profile through antioxidant, enzyme inhibition, and anticancer studies. Moreover, the computational approach will be followed to theoretically identify the interaction of the compounds with the studied compounds. This research will add valuable knowledge on the *A. heterophyllus* with highlights of biomedicinally significant compounds that could serve as therapeutic compounds.

## 2. Materials and Methods

### 2.1. Chemicals

Folin–Ciocalteu reagent, gallic acid, anhydrous sodium carbonate, quercetin, aluminum chloride, sodium nitrile, sodium hydroxide, tannic acid, ascorbic acid, 2,2-diphenyl-1-picrylhydrazyl (DPPH) solution, 2,2- azino-bis(3-ethylbenothiazoline) 6-sulfonic acid (ABTS), potassium per sulfate, DMSO, sulfuric acid, ammonium molybdate, sodium phosphate, acetate buffer, ferric chloride, hydrochloric acid, TPTZ, sodium nitroprusside, phosphoric acid, naphthyl ethylenediamine dihydrochloride, DPPH, sodium nitrile, sodium phosphate buffer, sodium chloride, dinitro salicylic acid, starch, ABTS, iodine reagent, acarbose, thiourea, urease, sulphanilamide, phosphate buffer, aqueous urea, phenol, sodium hypochloride, SK injection, L-dopa, tyrosinase enzyme, kojic acid, L-tyrosine, barium chloride, nutrient broth, agar media, water, methanol, and ethanol (analytical grade) were procured from Sigma-Aldrich, Louis, MO 63103, USA.

### 2.2. Extract Preparation

*Artocarpus heterophyllus* fruits were purchased from a farm in the city of Jazan, Saudi Arabia, in June 2022. These fruits were distinguished by Dr. Rajakrishnan Rajagopal at King Saud University, College of Science. 500 g of the fruit was collected, and then, the fruits were washed and cut into small pieces. Then, they were exposed to cold air to dry, followed by ground to produce a powder. After that, we soak each powder in 80% ethanol (250 mL x4), and then we stir frequently for 3 days and filter. The soaking process was repeated four times until all active compounds were extracted. After that, the alcoholic extracts were collected and evaporated on a rotary evaporator to obtain dark green residues that were kept in a dark vial in the refrigerator for further phytochemical and biological analyses.

### 2.3. Chemical Composition Evaluation

#### 2.3.1. Qualitative Screening of Phytoconstituents in EAHF

The qualitative preliminary phytochemical screening tests were carried out to detect the presence or absence of the primary and secondary metabolites such as amino acids, carbohydrates, lipids, proteins, reducing sugar, flavonoids, cardiac glycosides, phenols, saponins, tannins, steroids, and alkaloids in EAHF [10].

#### 2.3.2. Determination of Polyphenols

In the quantification of polyphenols, the total phenolic content (TPC), total flavonoid content (TFC), and total tannin content (TTC) were assessed in EAHF. The TPC was evaluated using the Folin–Ciocalteu method, as previously documented [11]. The results for TPC were expressed as milligrams of gallic acid equivalent per gram of dried extract. Similarly, the TFC in EAHF was evaluated following established protocols [12], with the findings reported as milligrams of quercetin equivalent per gram of dried extract. Additionally, the tannin contents were quantitatively estimated by employing the method of Folin–Ciocalteu [13], and the findings were computed in terms of mg TAE/g d.w. (milligrams of tannic acid equivalent per gram of dried extract).

#### 2.3.3. GC–MS Analysis

The extracted sample underwent analysis via GC–MS utilizing an Agilent 5977A GC system, which was equipped with an HP-5MS capillary column measuring 30 m in length, 250 μm in internal diameter, and 0.25 μm in film thickness, with a temperature tolerance of up to 350°C. This system was coupled with an Agilent 5977A Series mass selective detector (MSD). Ultrahigh purity helium (99.99%) served as the carrier gas and maintained at a constant flow rate of 1.2 mL/min. The temperatures for the injection port, transfer line, and ion source were consistently maintained at 310°C, with an ionization energy set at 70 eV. The oven temperature was programmed to increase from an initial 60°C, held for 7 min, to a final temperature of 310°C at a rate of 5°C/min. A 1 μL injection volume was employed with a split ratio of 50:1. Data acquisition involved the collection of full-scan mass spectra over a range of 35–650 atomic mass units (amu). The identification and characterization of chemical compounds were performed based on their GC retention times and the most closely matching fragmentation patterns. The mass spectra obtained were compared against standards from the NIST-02 mass spectrum libraries using the MassHunter GC/MS Acquisition software [14].

### 2.4. Evaluation of Biological Profile

#### 2.4.1. Antioxidant Activities

##### 2.4.1.1. Total Antioxidant Capacity (TAC) by Phosphomolybdenum Method

This antioxidant activity was performed according to the literature [14] with minor modifications. An ascorbic acid solution is taken as standard by dissolving 1 mg of ascorbic acid in 1 mL of 5% DMSO. Different concentrations of ascorbic acid 50–1000 μg/mL were prepared. After that standard test dilutions were prepared by dissolving 130 μL from each standard dilution of ascorbic and making the final volume up to 1 mL with phosphomolybdenum reagent in Eppendorf tubes. The test sample is prepared by mixing 130 μL of plant solution, and the final volume is made up to 1 mL with phosphomolybdenum reagent. Then, standard test dilutions and sample test solution were subjected to incubation at 95°C for 90 min. The readings of absorbance were measured at λ 695 nm. The results were represented as ascorbic equivalent per gram of (A.A. eq/g) dry weight of the extract.

##### 2.4.1.2. DPPH

The evaluation of antioxidant activity was performed following established methodologies [14], with slight modifications. A DPPH solution was prepared by dissolving DPPH in an appropriate solvent, typically methanol, to achieve a concentration of 0.3 mM. The antioxidant sample was prepared by dissolving or diluting it in a suitable solvent, also commonly methanol. An aliquot of the sample solution (90 μL) was combined with an equal volume of the DPPH solution (90 μL) in a microplate well. The mixture was then incubated in the dark at room temperature for a specified duration, generally between 30 min and 1 h, to allow for the reaction to occur. Following this incubation period, the absorbance of the resulting solution was measured at a specific wavelength, typically 517 nm, using a BioTek Synergy HT microplate reader. An ascorbic acid solution is taken as standard by dissolving 1 mg of ascorbic acid in 1 mL of 5% DMSO. The results were represented as ascorbic equivalent per gram of (A.A. eq/g) dry weight of the extract.

##### 2.4.1.3. ABTS

The evaluation of antioxidant activity was performed following established methodologies as previously documented [15], with minor adjustments. In particular, 1 mL of ABTS, 1 mL of potassium persulfate, and 1 mL of the sample solution were mixed using test tubes followed by an incubation period of 30 min in a light-deprived room. Absorbance was subsequently recorded at 620 nm using a BioTek Synergy HT microplate reader. The results were represented as ascorbic equivalent per gram of (A.A. eq/g) dry weight of the extract.

##### 2.4.1.4. Ferric Reducing Antioxidant Power (FRAP)

The FRAP assay was performed following a previously established methodology [15], with certain modifications implemented. A range of ascorbic acid concentrations (5–50 μg/mL) was utilized as standards in this assay. The extract solution (1 mg/mL) was prepared and taken in a quantity of 110 μL in a microtiter plate. Subsequently, a reaction mixture was prepared, consisting of 0.3 M acetate buffer (pH 3.6), 20 mM ferric chloride, 10 mM 2,4,6-tris(2-pyridyl)-s-triazine (TPTZ), and 40 mM HCl (70 μL). The mixture was incubated for 30 min, after which the absorbance was recorded at 593 nm using a BioTek Synergy HT microplate reader. The results were represented as ascorbic equivalent per gram of (A.A. eq/g) dry weight of the extract.

##### 2.4.1.5. Nitric Oxide Scavenging (NOS) Assay

The evaluation of NOS inhibition was performed using a previously established protocol [16], with certain modifications implemented. A range of sodium nitrite concentrations, specifically from 5 to 50 μg/mL, was utilized as standards. A solution (1 mg/mL) of the extract was prepared, from which 1 mL was aliquoted. Subsequently, 0.25 mL of sodium nitroprusside was incorporated into the aliquot, and the mixture was incubated for 2 h. Following incubation, 0.5 mL of the resultant solution was mixed with 0.3 mL of Griess reagent. The absorbance of the final solution was then measured at a wavelength of 570 nm using a BioTek Synergy HT microplate reader.

#### 2.4.2. Enzyme Inhibition Activities

##### 2.4.2.1. Tyrosinase Inhibition Assay

The tyrosine inhibitory activity has been carried out by the procedure given in the literature [17] with minor modifications. 20 μL of tyrosinase enzyme, 10 μL of assay compound, and 150 μL of phosphate were added in each well and subjected to incubation for 10 min followed by measurement of absorption at 480 nm which was noted as preread. Add 20 μL L-dopa and incubate for 30 min. Then, the afterread was taken at the wavelength of 480 nm. Kojic acid was used as positive control, and methanol was used as negative control. Percentage inhibition was calculated by the formula mentioned in the literature.

##### 2.4.2.2. α-Amylase Inhibition Assay

The effect of EAHF on α-amylase inhibition was assessed by employing the starch–iodine test following the previously documented protocols [14]. The experimental protocol involved the incubation of 100 μL of 0.02 M sodium phosphate buffer, 100 μL of NaCl, 100 μL of soluble starch, and 100 μL of plant acarbose/extract at a temperature of 37°C for 5 min. Acarbose was employed as the standard reference. Following this initial incubation, 195 μL of amylase solution was added and the mixture was incubated at 37°C for an additional 10 min. The reaction was subsequently halted by the addition of 260 μL of dinitrosalicylic acid followed by the addition of iodine reagent in a quantity of 100 μL, and any resulting color change was recorded. The absorbance was measured at a wavelength of 620 nm, and the percentage inhibition was calculated using the formula outlined in the relevant literature.

##### 2.4.2.3. Urease Inhibition Assay

The urease inhibition potential of EAHF was assessed using a method reported in the literature [18] with some modifications. A mixture of 10 μL of urease solution, 10 μL of phosphate buffer pH 7, and 10 μL of sample solution was added to the microplate well, followed by incubation for 15 min. 30 μL aqueous urea was added to the reaction mixture followed by incubation for 15 min. Prereading was taken at 630 nm followed by the addition of alkali reagent (50 μL) followed by the addition of 30 μL of phenol reagent to the mixture. Then, the mixture is set at an incubation period of 90 min. Then, at 630 nm, absorbance was measured. Percentage inhibition was calculated by the formula mentioned in the literature.

##### 2.4.2.4. Thrombolytic Activity

The thrombolytic activity was evaluated following established protocols [14]. Blood samples were collected in incubated Eppendorf tubes, from which serum was carefully separated without disrupting the blood clot. The clots were subsequently placed back into the tubes, which were then reweighed. Then, 100 μL from the extract solution (1 mg/mL) was added along with SK serving as the standard reference to each Eppendorf tube which was already having a blood clot. The samples were then subjected to incubation for 1.5 h at 37°C. Following this incubation period, the fluid that had been released into the Eppendorf tubes was removed and the tubes were reweighed. The thrombolytic potential was calculated using the formula documented in the literature.

#### 2.4.3. Hemolytic Activity

The hemolytic activity was assessed following established methodologies documented in the literature [14]. This activity is indicative of the initial toxicity of the plant extract on human erythrocytes. Blood samples were collected from a healthy donor and subsequently centrifuged at 4000 rpm for 15 min, after which the plasma was meticulously removed. The remaining erythrocytes were then washed with a phosphate buffer solution having pH of 7.4. A mixture consisting of the plant extract (975 μL) and erythrocytes (25 μL) was prepared and incubated for 1.5 h at 37°C. Following the incubation period, the mixture was centrifuged for 3 min at 2000 rpm to facilitate the separation of the supernatant, from which hemolysis was evaluated by recording the absorbance at 540 nm. The positive control used in this analysis was a 0.1% solution of Triton X-100, while the negative control used was phosphate-buffered saline.

#### 2.4.4. Cytotoxicity Assay

Triple-negative breast cancer cell lines (MDA-MB-231) and MCF7 were cultured in Dulbecco's Modified Eagle Medium (DMEM) enriched with 10% fetal bovine serum. The cultures were maintained at a temperature of 37°C in an atmosphere containing 5% carbon dioxide and 90% relative humidity. The cytotoxic effects of various test substances were evaluated using a standard MTT-based colorimetric assay. Upon achieving 80% confluence, the monolayer cells were subcultured through trypsinization and subsequently plated in 96-well plates at a density of 2 × 10^4 cells per well, utilizing 100 μL of cell suspension in a complete medium. Following a 24-h incubation period, cells that demonstrated exponential growth were subjected to treatment with either 100 μL of fresh medium (control) or medium supplemented with the test samples. After an additional 24 h, the cells were rinsed with phosphate-buffered saline (PBS, pH 7.4) and treated with 100 μL of 0.5 mg/mL MTT in PBS, followed by a 3-h incubation. The incubation was extended by the addition of 100 μL of dimethyl sulfoxide (DMSO). The optical absorbance of the MTT solution above the cell layers was subsequently measured at a wavelength of 550 nm. The standard drug doxorubicin (dox) at a concentration of 5 mcg/mL was regarded as the positive control in this experiment. The percentage of cell viability was calculated using a specific equation, and the cytotoxic activity of the test materials was quantified in terms of IC50 values [19].

(1) $$\begin{array}{c} \text{Cell viability }\left(\%\right)=\frac{\left(Ab550\text{ sample}\times 100\right)}{Ab550\text{ control}}. \end{array}$$

### *2.5.* In Silico Activities

#### 2.5.1. Molecular Docking

It is a highly helpful tool for studying drug design with computer assistance. First, PDB formatted protein resolutions for urease (2Kau), tyrosinase (2y9x), and alpha-amylase (1bli) were acquired from the Protein Data Bank (PDB). The protein preparation for the Discovery Studio 2021 Client was finished. Protein molecules had various chains deleted, except the A chain, water molecules, and heteroatoms that were previously connected. Protein molecules were then combined with polar hydrogen molecules and stored as a PDB file. Standard compounds were retrieved in structure data file (SDF) format from the PubChem database and secondary metabolites were selected based on the GC–MS analytical method table. After that, the macromolecule option was completed and the created protein molecule was submitted to the PyRx program. Then, using the openbabel option, ligands were uploaded into PyRx. These ligands were then reduced in size and transferred to PDBQT format. Subsequently, a grid box with precise dimensions was created, and the ligand–protein interaction was initiated to examine the binding outcomes. Ultimately, Discovery Studio was used to depict the interactions.

#### 2.5.2. Absorption, distribution, metabolism, and excretion (ADME) Analysis

The ADME profile of the selected was evaluated using an in silico approach with the help of the online SwissADME tool https://www.swissadme.ch/accessed on 11-02-2024.

#### 2.5.3. Toxicity Evaluation

The in silico toxicity parameters of the selected compounds were docked with the help of the online program PROTOX II https://tox-new.charite.de/accessed on 11-02-2024.

### 2.6. Statistical Analysis

All experiments were conducted in triplicate, and the results are presented as mean ± standard deviation. To assess statistical significance, one-way ANOVA followed by Tukey's test was employed, utilizing GraphPad Prism 7.0 software. A *p* value of less than 0.05 was deemed statistically significant.

## 3. Results

### 3.1. Phytochemical Composition of EAHF

#### 3.1.1. Preliminary Phytochemical Screening

Various qualitative phytochemical screening tests were used to identify the phytoconstituents in EAHF.  Table 1 shows the primary and secondary metabolites (i.e., phenols, carbohydrates, glycosides, tannins, alkaloids, and amino acids are present in the EAHF). A preliminary phytochemical analysis was conducted on EAHF. This investigation revealed that proteins and lipids were absent from the extracts; however, a large number of primary and secondary metabolites were found to be present in EAHF. Nonetheless, a small percentage of carbohydrate content was found. The EAHF was observed to have certain amounts of amino acids. It was found that EAHF containing tannins and phenols included alkaloids as secondary metabolites. The EAHF showed no signs of flavonoids or saponins. The assay reveals the presence of resins, glycosides, and cardiac glycosides while excluding steroids.

#### 3.1.2. Polyphenolic Contents in EAHF

The TPC of EAHF was calculated as recorded to be 115.5 ± 5 mg of gallic acid equivalent per gram of dry extract. TFC was (77 ± 2.22 mg Qu.E/g extract) and TTC was (59.33 ± 1.66 mg Qu.E/g extract). Results are expressed in Table 2.

#### 3.1.3. GC–MS

**Figure 1:**
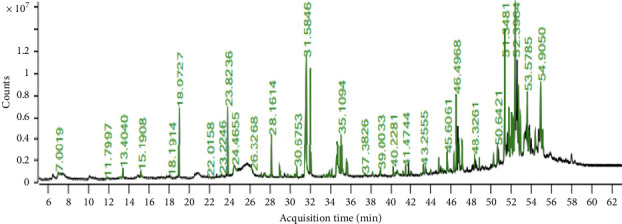
GC–MS chromatogram of EAHF.

The GC–MS chromatogram of EAHF showed 81 peaks of phytocompounds. Major phytocompounds tentatively identified in the GC–MS were consisting of pregn-4-ene-3,20-dione, 16,17-epoxy-, (16.alpha), 24-norursa-3,12-diene, lanosta-8,24-diene-3,22-diol, lupeol, 7-dehydrodiosgenin, lup-20(29)-en-3-one, alpha-amyrin, 23-(phenylsulfanyl)lanosta-8,24-dien-3-ol, 12-oleanen-3-yl acetate,(3.alpha.), and 9,19-cyclolanost-23-ene-3,25-diol, (3.beta.,23E)-. Majority of the compounds were belonged primarily to the chemical classes of alkanes followed by steroids, fatty acids, triterpenoids and esters. Details of the phytocompounds identified by GC–MS are given in Table 3, and peaks are shown in Figure 1.

### 3.2. Biological Profiling

#### 3.2.1. Antioxidant Potential of EAHF

The antioxidant capacity of EAHF was evaluated utilizing the FRAP, DPPH, TAC, NOS, and ABTS methodologies. The findings of these antioxidant assessments are summarized in Table 4. The DPPH assay indicated that EAHF demonstrated significant activity, yielding a value of 204.90 ± 4.90 mg TE/g D. E. These results suggest that EAHF possesses considerable antioxidant potential. Furthermore, numerous phytocompounds with known antioxidant properties were identified in EAHF through GC–MS analysis. In the FRAP evaluation, the extract exhibited a maximum reduction potential of 258.36 ± 1.84 mg TE/g D. E. The ABTS assay revealed an activity level of 159.5 ± 5 mg TE/g D. E, while the TAC and NOS assays produced results of 116.87 ± 0.62 mg TE/g D. E and 93.36 ± 1.04 mg TE/g D. E, respectively. Additionally, the GC–MS analysis corroborated the presence of various phytocompounds with antioxidant capabilities. Notably, the elevated levels of TFC and TPC in EAHF may significantly enhance its antioxidant activities.

#### 3.2.2. Enzyme Inhibition Activities

The enzyme inhibition properties of EAHF were evaluated for its potential applications in cosmetics, specifically as a skin-lightening agent through tyrosinase inhibition, as well as for its antidiabetic effects via α-amylase inhibition and antiulcer activity through urease inhibition. Acarbose, kojic acid, and thiourea were utilized as standards for α-amylase, tyrosinase, and urease inhibition, respectively, to assess the enzyme inhibition potential of EAHF. The results indicated that EAHF demonstrated an α-amylase inhibitory effect of 28.17%, in contrast to the standard acarbose, which exhibited an inhibition rate of 97.16%. In terms of tyrosinase activity, EAHF showed an inhibitory effect of 71.01%, compared to the standard kojic acid, which had an inhibition rate of 97.82%. Furthermore, the urease activity results revealed that EAHF exhibited a urease inhibitory effect of 95.65%, while the standard thiourea showed an inhibition rate of 94.20%. These findings are summarized in Table 5.

#### 3.2.3. Thrombolytic and Hemolytic Activity

EAHF showed significant thrombolytic activity with a value of 85.51% compared to standard which has shown activity of 99.35%. The findings are displayed in Table 6. In the case of hemolytic activity, there was a significant effect observed on the hemolysis. EAHF has shown only 1.01% hemolytic, while the standard was found with 90.90% hemolytic activity as shown in Table 6.

#### 3.2.4. Anticancer Potential

**Figure 2:**
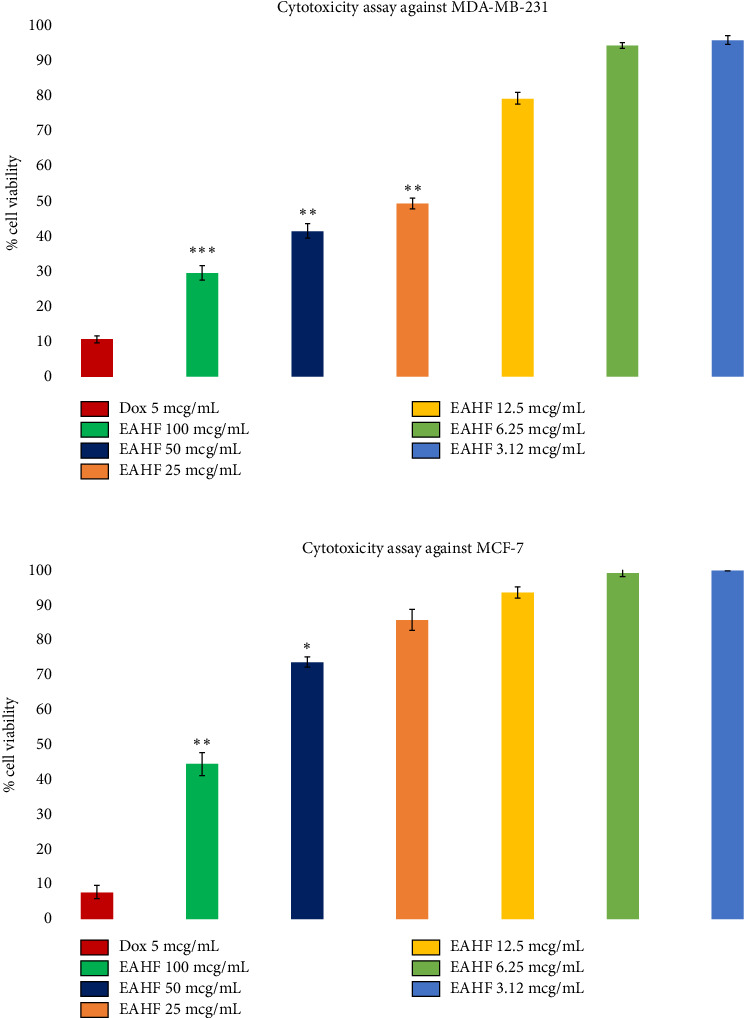
The cytotoxicity evaluation of EAHF against breast cancer cell lines (MDA-MB-231 and MCF-7). All the values are expressed as mean ± standard deviation (*n* = 3). The data were analyzed by using GraphPad Prism software (V: 9.0) by applying one-way ANOVA followed by Tukey's multiple comparison test. The symbols used for denoting the significance are ^∗^ when *p* value ≤ 0.05, ^∗∗^*p* ≤ 0.01, and ^∗∗∗^*p* ≤ 0.001.

In breast cancer evaluation, the extract showed an antiproliferative effect against breast cancer cell lines MDA-MB-231 and MCF-7 in a concentration-dependent manner.  Figure 2 shows the findings of the study, revealing a higher anticancer effect of EAHF against MDA-MB-231 compared to MCF-7. The maximum effects were observed at 100 mcg/mL (*p* ≤ 0.001) and *p* ≤ 0.01 in MDA-MB231 and MCF-7 cell lines, respectively, with IC_50_ values of 17.78 μg/mL against MDA-MB231 cell lines.

### 3.3. In Silico Studies

#### 3.3.1. Molecular Docking

Molecular docking studies were conducted on all phytocompounds identified through GC–MS concerning the enzymes α-amylase, tyrosinase, and urease (Table 1S of Supporting information file). The docking analysis indicated that the binding affinities of 23 phytocompounds surpassed that of acarbose, the standard reference, in the case of α-amylase. Among the tested phytocompounds, 7-dehydrodiosgenin demonstrated the highest activity, exhibiting a binding affinity of −12.8 kcal/mol. This compound interacted with the active site of α-amylase by forming van der Waals interactions with the amino acid residues ASP164, THR163, TYR198, GLY108, THR49, and ALA109. The 2D structural analysis of the ligands concerning α-amylase revealed that pregn-4-ene-3,20-dione, 16,17-epoxy-, (16.alpha), lanosta-8,24-diene-3,22-diol, Lup-20(29)-en-3-one, alpha-amyrin, and 9,19-cyclolanost-23-ene-3,25-diol, (3.beta.,23E) exhibited hydrogen bond interactions. Conversely, 24-norursa-3,12-diene, lupeol, 12-oleanen-3-yl acetate, (3.alpha.), and 23-(phenylsulfanyl)lanosta-8,24-dien-3-ol, along with 7-dehydrodiosgenin, did not demonstrate any hydrogen bond interactions.

**Figure 3:**
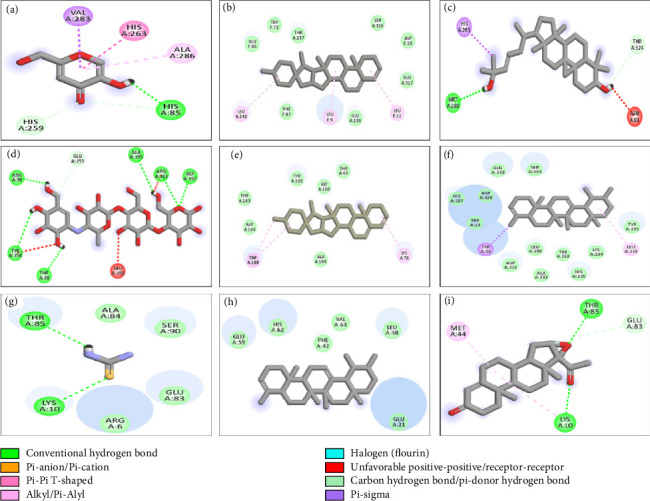
The interaction of docked compounds at the active site of tyrosinase. (a) Kojic acid (standard), (b) 7-dehydrodiosgenin, and (c) 9,19-cyclolanost-23-ene-3,25-diol, (3.beta.,23E); the active site of amylase (d) acarbose (standard), (e) 7-dehydrodiosgenin, and (f) 24-norursa-3,12-diene; and the active site of urease (g) thiourea (standard), (h) 24-norursa-3,12-diene, and (i) pregn-4-ene-3,20-dione, 16,17-epoxy-, (16.alpha.).

In the case of tyrosinase, the docking results indicated that 7-dehydrodiosgenin also exhibited the highest binding affinity of −11.1 kcal/mol, interacting with the active site of tyrosinase through van der Waals interactions with the amino acid residues GLU86, TRP72, THR237, SER316, ASP10, GLU317, GLU239, and PHE87. The 2D structural analysis of the ligands with tyrosinase revealed that pregn-4-ene-3,20-dione, 16,17-epoxy-, (16.alpha.), lanosta-8,24-diene-3,22-diol, alpha-amyrin, 23-(phenylsulfanyl)lanosta-8,24-dien-3-ol, 12-oleanen-3-yl acetate, (3.alpha.), and 9,19-cyclolanost-23-ene-3,25-diol, (3.beta.,23E) exhibited hydrogen bond interactions, while 24-norursa-3,12-diene, lupeol, lup-20(29)-en-3-one, and 7-dehydrodiosgenin did not. For urease, the docking results indicated that 7-dehydrodiosgenin exhibited the highest binding affinity of −10.9 kcal/mol, interacting with the active site of urease through van der Waals interactions with the amino acid residues TYR32, PRO444, SER436, LYS124, VAL471, and GLN469, as well as hydrogen bond interactions with LYS443. The 2D structural analysis of the ligands with urease showed that pregn-4-ene-3,20-dione, 16,17-epoxy-, (16.alpha.), lanosta-8,24-diene-3,22-diol, lup-20(29)-en-3-one, alpha-amyrin, 7-dehydrodiosgenin, and 9,19-cyclolanost-23-ene-3,25-diol, (3.beta.,23E) exhibited hydrogen bond interactions, while 24-norursa-3,12-diene, lupeol, 12-oleanen-3-yl acetate, (3.alpha.), and 23-(phenylsulfanyl)lanosta-8,24-dien-3-ol did not. The binding results of all phytocompounds against tyrosinase, α-amylase, and urease are summarized in Table 1S, while the 2D and 3D structures illustrating the interactions of compounds with the highest binding affinities are presented in Figure 3. Overall, the results of docking analysis support the conclusion that the in vitro enzyme inhibitory activities of the extract might be due to the presence of the said phytoconstituents that have shown good docking interaction with the enzymes. Furthermore, these findings provide a direction to isolate these compounds and evaluate against these enzymes using in vitro models.

#### 3.3.2. ADME Analysis

**Figure 4:**
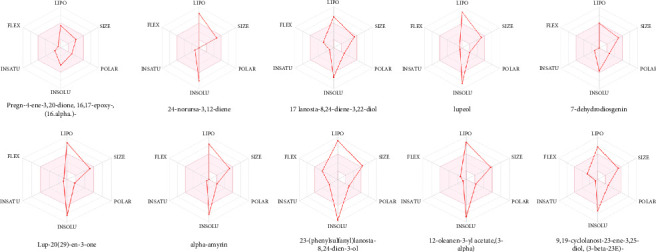
Bioavailability radar of phytocompounds exhibiting maximum binding results.

A total of 10 phytoconstituents identified from the EAHF GC–MS analysis were selected based on their maximum binding affinity for ADME. The Swiss ADME tool provides insights into the physicochemical properties, pharmacokinetics, and drug-likeness characteristics of these compounds. Phytoconstituents that do not comply with two or more of Lipinski's Rule of Five are typically considered unsuitable for oral administration. The ADME analysis of the selected phytoconstituents from EAHF indicated that all but one of the compounds violated at least one of the rules, except for 23-(phenylsulfanyl)lanosta-8,24-dien-3-ol, which violated two rules, as detailed in Table 7. The majority of the selected and analyzed phytoconstituents were deemed appropriate for oral administration and exhibited characteristics indicative of orally active drug-likeness, except for one compound. The oral drug delivery system offers significant advantages, including enhanced safety, pain avoidance, and improved patient compliance, compared to alternative delivery methods. Table 7 outlines various physicochemical parameters, including pharmacokinetic behavior, lipophilicity, molecular weight, number of bond rotations, and the number of hydrogen bond acceptors and donors for the selected phytoconstituents.  Figure 4 illustrates the bioavailability radar for the selected phytoconstituents from EAHF. Notably, there is a lack of existing literature concerning the ADME analysis of EAHF.

#### 3.3.3. Toxicity Analysis

In this analysis, 10 phytoconstituents from EAHF GC–MS analysis were selected based on maximum binding affinity. PROTOX ll provides information about the hepatotoxicity, predicted LD_50,_ carcinogenicity, predicted toxicity class, mutagenicity, cytotoxicity, and immunotoxicity of the phytoconstituents. All the phytoconstituents show positive results in immunotoxicity. Only pregn-4-ene-3,20-dione, 16,17-epoxy-, (16.alpha)-, and 12-oleanen-3-yl acetate,(3.alpha.) show positive results in carcinogenicity. All the phytoconstituents fall in toxicity Classes 4, 5, and 6. There is no reported literature available regarding the toxicity analysis of EAHF. Results are expressed in Table 8.

## 4. Discussion

This research provides comprehensive insights into the phytochemical composition of EAHF. The chemical profiling indicates that EAHF is a significant source of glycosides, alkaloids, phenols, carbohydrates, tannins, and amino acids. These phytochemical classes are recognized for their antioxidant, antifungal, anticancer, antidiabetic, antimicrobial, anti-inflammatory, and antihypertensive properties [20]. Specifically, alkaloids exhibit antioxidant, antitumor, and antidiabetic effects, while phenols and tannins are noted for their antioxidant and anticancer capabilities. The presence of these phytochemicals in EAHF suggests potential therapeutic applications [21]. The genus is particularly noteworthy due to its high levels of total bioactive compounds. The elevated TPC and TFC in EAHF are associated with its pronounced antioxidant activities. For the identification of phytochemicals, EAHF was analyzed using GC–MS, which revealed 81 distinct peaks corresponding to various compounds. The predominant compounds identified through GC–MS include pregn-4-ene-3,20-dione, 16,17-epoxy-, (16.alpha)-, 24-norursa-3,12-diene, lanosta-8,24-diene-3,22-diol, lupeol, 7-dehydrodiosgenin, lup-20(29)-en-3-one, alpha-amyrin, 23-(phenylsulfanyl)lanosta-8,24-dien-3-ol, 12-oleanen-3-yl acetate, and 9,19-cyclolanost-23-ene-3,25-diol. The primary chemical class identified in EAHF was alkanes, followed by fatty acids, steroids, fatty acid esters, and phenolic compounds, with esters also being present. Furthermore, GC–MS analysis uncovered numerous secondary metabolites with diverse biological activities, including anti-inflammatory, antioxidant, and antidiabetic properties, as detailed in Table 3. The phytochemical analysis of EAHF underscores the presence of bioactive compounds, positioning it as a promising source for the isolation of novel molecules for drug discovery.

On this basis, we decided to examine cytotoxicity, antioxidant, and antidiabetic activities. The results of the FRAP, DPPH, TAC, ABTS, and NOS activities are higher than those observed in the literature [22] showing that there is a correlation between antioxidant potential and the polyphenol contents (i.e., high level of flavonoid and phenolic contents correlates with the strong antioxidant potential). Flavonoids are polyphenolic compounds naturally found in most edible vegetable and fruit plants. They constitute mostly yellow, blue, and red colors in the fruit [23]. Phenolic compounds are the most significant types of secondary metabolites in plants. The quality and quantity of TPC and other secondary metabolites biosynthesized in natural products vary massively [17].

ROS, which include HO_2_, OH, and OONO (apart from O_2_), are often generated during metabolic activities, and their excess can have detrimental effects on proteins, DNA, and fatty acids resulting in inflammation and tissue damage. Consequently, consuming large amounts of antioxidants strengthens the immune system by scavenging or detoxifying these free radicals. The linear regression equation was plotted to calculate the scavenging potential of the various solvent extracts [18]. Additionally, the presence of compounds with anti-inflammatory and antioxidant qualities may aid in the treatment of skin disorders. Tyrosine is needed for the creation of melanin. Tyrosinase overexpression and elevated melanin are the causes of age-related wrinkles. In the food and cosmetics industries, antioxidants and tyrosinase inhibitors are considered as preservatives and skin-protecting chemicals [24]. Although there have been several skin-whitening products released on the market in recent decades, these treatments have not yet been shown to be effective. As demonstrated by hydroquinone, this typically occurs as a result of the elevated toxicity and mutagenesis effects of these bleaching agents [25]. As it became clear that new natural tyrosinase inhibitors with enhanced skin penetration, higher therapeutic efficacy, and low side effects were being sought after, the significance of the study design increased. Table 5 revealed that EAHF had respectable antityrosinase efficacy. The results shown in this study [26] show that *A. heterophyllus* stem bark and peel extracts are the most promising source for skin-lightening agents due to their substantial melanin content reduction effect, nontoxicity, and potent cellular tyrosinase inhibitory potential.

Bioactive compounds such as polyphenols, which may also be lowering oxidative stress, may be responsible for the extracts' antidiabetic properties. GC–MS analysis was used to infer many phytoconstituents with known antidiabetic activities. These phytocompounds may be impacting EAHF activity. Additionally, the higher polyphenol content may be enhancing EAHF's action due to a possible synergy with terpenoids and other nonpolar components. Diabetes mellitus, one of the most common endocrine metabolic illnesses, has been associated with significant morbidity and mortality due to microvascular effects (neuropathy, nephropathy, and retinopathy) and macrovascular repercussions (peripheral vascular disease and heart stroke) [27]. EAHF shows inhibitory potential for alpha-amylase as stem bark of A. Heterophyllus has the inhibitory potential of alpha-amylase which was mediated by noncompetitive and uncompetitive, respectively [28].

The urease enzyme plays a critical role in the long-term colonization of Helicobacter pylori (*H. pylori*), which causes gastrointestinal disorders, including duodenal, peptic, and gastric cancer. For a considerable amount of time, plants have been used as the primary source of naturally occurring compounds with therapeutic characteristics, which reduces their toxicity and negative side effects when used [29]. Roughly, 4.6 million Americans have peptic ulcers; 10% of those individuals also have duodenal ulcers. 90% of duodenal ulcers and 70 percent of peptic ulcers are caused by infection caused by Helicobacter pylori. The mucosal epithelium of the stomach can be adversely affected by *H. pylori*'s extracellular urease secretion [18]. It is commonly known that *H. pylori* secrete urease, an enzyme that contains nickel and converts urea into ammonia (NH3), shield the bacteria from the stomach's acidic environment. One effective way to prevent *H. pylori* infections is to use urease inhibitors, which can regulate the activity of urease. Infections brought on by bacteria that produce urease can be effectively eradicated with the use of urease inhibitors. As particular urease inhibitors in this situation, several inorganic salts, heavy metal ions, synthetic chemical compounds, and antibiotics have been employed [30]. The antiurease activity of EAHF is very high a little more than standard, and to our knowledge, there is no work reported to antiurease activity in the literature.

It has been found that plants with flavonoids and polyphenols have thrombolytic action. EAHF exhibited significant thrombolytic action. Staphylokinase and SK are examples of microbial plasminogen activators that work as cofactoring molecules to support development. Red blood cells release their hemoglobin when the RBC membrane breaks down or becomes disrupted, a process known as hemolysis. When utilized for a lengthy length of time, certain traditional plants can occasionally become hazardous. Numerous plants have chemical components that might either hemolysis or have an antihemolytic impact on human erythrocytes. Red blood cell membranes can be damaged by plant extracts, which can have major adverse effects, i.e., hemolytic anemia. It is important to assess the hemolytic potential of herbs. When hemolysis exceeds 30%, the extracts are considered detrimental to erythrocytes. The hemolytic activity of EAHF is presented in Table 6, with hemolysis quantified as a percentage. The findings indicate that the extract exhibited a hemolytic potential of 1.01%, which is below the 30% threshold, thereby categorizing it as nontoxic and safe for human consumption.

Hemostasis failure is a big problem these days. It leads to the formation of thrombus, or blood clots, which can completely or partially block small blood vessels in the circulatory system. Catastrophic thrombotic diseases, i.e., acute myocardial infarction and cerebral infarction, can arise due to arterial occlusion and ultimately lead to death. Treatment for thrombus often involves the use of thrombolytic drugs, i.e., urokinase, tissue plasminogen activator, alteplase, anistreplase, and SK. They are mostly artificial and have bad qualities. Investigating local resources for cutting-edge, secure, and effective therapies that have substantial thrombolytic action is therefore essential [31]. This is the purpose of finding the thrombolytic activity of EAHF. We have observed a significant thrombolytic activity of the extract (85.5%).

Breast cancer is marked as the second leading cancer after lung cancer in terms of prevalence and mortality rate. The currently available treatment options for breast cancer include surgery, radiation, and chemotherapy. Surgery has been observed with notable success; however, it is associated with recurrence and damage to the quality of life of the patients. Similarly, the use of radiation is also associated with some life-threatening side effects including mutation of various genes and lifetime disability [32, 33]. The chemotherapeutic agents have proved vital in the treatment of breast cancer, but the clinical implications are limited due to the associated side effects. Dox is the most commonly used anticancer drug in the treatment of breast cancer. However, it is reported to cause cardiotoxicity, neurotoxicity, and bone marrow depression. These side effects badly affect the patient's compliance rate which necessitates the need for the search of new therapeutic alternatives with antibreast cancer potential [34]. In this regard, natural products are found with dynamic potential against various types of cancer with minimum side effects [35]. Recently, an increase in research on medicinal plants has been observed with the aim of identification and discovery of medicinal compounds with anticancer potential. In this study, we reported the antibreast cancer potential of EAHF using MTT cytotoxicity assay. It was found that the extract possesses activity against MCF-7 and MDA-MB-231 cell lines in a concentration and time-dependent manner (Figure 3). The extract was more active toward MDA-MB-231 compared to MCF-7, which is very interesting because the treatment of MDA-MB-231 is more challenging than MCF-7.

A compound's total cytotoxicity against normal, healthy cells can be determined by looking at its hemolytic activity. Plants have phytochemicals called saponins that produce hemolytic action when they contact with the membranes of red blood cells. Quantifying hemolysis is made easy and effective by the spectroscopic method used in the in vitro hemolytic test. The effects of various biomolecule concentrations on human erythrocytes may be assessed using this technique [4]. According to our results, the extracts show no hemolytic activity which is correlated with previous reports where the seed extract of the jackfruits [36] shows no hemolytic activity.

The integration of in vitro enzymatic assays with in silico molecular docking and absorption, distribution, metabolism, excretion, and toxicity (ADMET) profiling has emerged as a powerful approach for the early-stage evaluation of bioactive compounds [37]. This multidisciplinary strategy is particularly relevant when screening phytochemical-rich extracts, such as those characterized via GC–MS, for potential therapeutic applications [38, 39]. To assess the capacity of the compound to inhibit enzymes and to examine the correlation between the in vitro enzyme inhibition results, all phytocompounds identified through the GC–MS analysis of EAHF were subjected to docking studies against the enzymes α-amylase, tyrosinase, and urease, in addition to acarbose, kojic acid, and thiourea. The formation of hydrogen bonds and other hydrophobic interactions, including pi-alkyl and alkyl interactions, are essential for the stable binding of ligands to proteins and for the overall protein–ligand interactions. For further investigation, phytocompounds exhibiting the highest binding affinity were analyzed using SwissADME, which provides insights into their drug-likeness, pharmacokinetics, and physicochemical properties. The ProTox-II tool is utilized to assess compounds with established toxicological profiles and to generate toxicity predictions based on structural similarities [39]. In this study, 10 phytoconstituents identified from the EAHF GC–MS analysis were selected based on their maximum binding affinity and subsequently evaluated for toxicity using the ProTox software. This software provides insights into various toxicity parameters, including the predicted toxicity class, predicted LD50, mutagenicity, hepatotoxicity, carcinogenicity, cytotoxicity, and immunotoxicity of the selected phytoconstituents. Notably, all phytoconstituents exhibited positive results for immunotoxicity. Furthermore, only pregn-4-ene-3,20-dione, 16,17-epoxy-, (16.alpha)-, and 12-oleanen-3-yl acetate, (3.alpha.) demonstrated positive results for carcinogenicity. The phytoconstituents were classified within toxicity Classes 4, 5, and 6. It is important to note that there is a lack of existing literature concerning the toxicity analysis of EAHF. Overall, by combining in vitro enzyme inhibition assays with in silico docking and ADMET profiling, this study seeks to comprehensively evaluate the therapeutic relevance of GC–MS–identified phytochemicals in the extract. This integrated approach not only supports mechanistic interpretations but also aids in the identification of promising enzyme inhibitors with favorable pharmacological profiles for future drug development.

## 5. Conclusions

The current study provided valuable insights into the phytochemical and pharmacological potential of the Saudi cultivar of jackfruits (*A. heterophyllus*) which was recently introduced for cultivation in Saudi Arabia. This study revealed that EAHF contained pharmacologically important phytochemicals that possess promising antioxidant, antibacterial, and enzyme (tyrosinase, urease, and α-amylase) inhibition potential. The EAHF's GC–MS analysis revealed the identification of the compounds. The inhibitory potentials of EAHF enzymes were further substantiated by in silico molecular docking studies. The extract showed multifaceted pharmacological activities including anticancer. The study indicated that the extract can be utilized for the isolation of novel compounds that will be used to treat disorders that involve these targeted enzymes. Overall, the findings support the traditional use of the species in various common ailments. However, further in-depth studies are required to further rectify the therapeutic utilization of the extract. Furthermore, in vivo toxicological research may be conducted to evaluate the EAHF's potential for use in diabetes, inflammatory diseases, and skin conditions.

## Figures and Tables

**Table 1:** Preliminary phytochemical profiling of EAHF.

Metabolite	Test	EAHF
Amino acids	Ninhydrin test	+
Carbohydrates	Molisch's reagent	+
Lipids	Saponification test	−
Protein	Biurette test	−
Reducing sugar	Fehling's test	+
Cardiac glycosides	Keller Kiliani test	+
Flavonoids	Ferric chloride test	−
Phenols	Ferric chloride test	+
Saponins	Froth test	−
Steroids	Salkowski's test	−
Tannins	Lead acetate test	+
Alkaloids	Hager's test	+

*Note:* present; “+,” absence; “−.”

**Table 2:** Polyphenolic contents of EAHF.

Sample	TPC	TFC	TTC
EAHF	115.5 ± 5	77 ± 2.22	59.33 ± 1.66

*Note:* All the results are expressed in mean ± standard deviation.

**Table 3:** Details of phytoconstituents identified in EAHF through GC–MS analysis.

Peak no	Retention time	Area	Compound name	Mol. weight	Mol. formula	Class
1	7.00	0.30	Hexanoic acid	116.16	C_6_H_12_O_2_	Carboxylic acids
2	11.79	0.12	4H-pyran-4-one, 2,3-dihydro-3,5-dihydroxy-6-methyl-	144.12	C_6_H_8_O_4_	Pyranones
3	13.1369	0.08	D-mannose	180.16	C_6_H_12_O_6_	Monosaccharides
4	13.40	0.37	Dodecane	170.33	C_12_H_26_	Alkanes
5	15.19	0.37	2-Decenal, (E)-	154.25	C_10_H_18_O	Aldehydes
6	18.19	0.03	Tridecane, 3-methyl-	198.39	C_14_H_3_O	Alkanes
7	18.97	1.82	Tetradecane	198.39	C_14_H_3_O	Alkanes
8	22.01	0.04	2(4H)-benzofuranone, 5,6,7,7a-tetrahydro-4,4,7a-trimethyl-, (R)-	180.24	C_11_H_16_O_2_	Lactones
9	23.22	0.04	1H-cycloprop[e]azulen-7-ol,decahydro-1,1,7-trimethyl-4-methylene-,(1ar-[1a.alpha.,4a.alpha.,7.beta.,7a.beta.,7b.alpha.])-	220.35	C_15_H_24_O	Sesquiterpenoids
10	23.82	1.80	Hexadecane	226.44	C_16_H_34_	Alkanes
11	24.46	0.92	1,3,4,5-Tetrahydroxy cyclohexane carboxylic acid	192.17	C_7_H_12_O_6_	Polyhydroxy carboxylic acids
12	26.32	0.05	9-Octadecen-1-ol, (Z)-	268.5	C_18_H_36_O	Alcohols
13	27.14	0.06	Pentadecane, 8-hexyl-	296.6	C_21_H_44_	Alkanes
14	27.38	0.07	6-Hydroxy-4,4,7a-trimethyl-5,6,7,7a-tetrahydrobenzofuran-2(4H)-one	196.24	C_11_H_16_O_3_	Lactones
15	27.47	0.12	Tetradecanoic acid	228.37	C_14_H_28_O_2_	Fatty acids
16	27.55	0.08	Heptadecane, 3-methyl-	254.5	C_18_H_38_	Alkanes
17	28.03	0.07	Tetradecanoic acid, ethyl ester	256.42	C_16_H_32_O_2_	Esters
18	28.16	1.11	Octadecane	254.5	C_18_H_38_	Alkanes
19	28.94	0.24	Neophytadiene	278.5	C_20_H_38_	Sesquiterpenoids
20	29.05	0.16	2-Pentadecanone, 6,10,14-trimethyl-	268.5	C_18_H_36_O	Ketones
21	29.79	0.09	Phytol acetate	338.6	C_22_H_42_O_2_	Acetates
22	30.06	0.03	Pentadecanoic acid, ethyl ester	270.5	C_17_H_24_O_3_	Esters
23	30.44	0.05	7,9-Di-tert-butyl-1-oxaspiro(4,5)deca-6,9-diene-2,8-dione	276.4	C_17_H_24_O_3_	α, β - unsaturated ketone
24	30.55	0.07	Z-(13,14-epoxy)tetradec-11-en-1-ol acetate	268.39	C_16_H_28_O_3_	Epoxy esters
25	30.67	0.26	Hexadecanoic acid, methyl ester	270.5	C_17_H_34_O_2_	Fatty acids methyl esters
26	31.16	0.04	Octadecane, 2-methyl-	268.5	C_19_H_4_O	Alkanes
27	31.58	9.04	n-Hexadecanoic acid	256.42	C_16_H_32_O_2_	Fatty acids
28	31.98	2.82	Hexadecanoic acid, ethyl ester	284.5	C_18_H_36_O_2_	Esters
29	32.09	0.56	Eicosane	282.5	C_20_H_42_	Alkanes
30	33.33	0.11	Heptadecanoic acid	270.5	C_17_H_34_O_2_	Fatty acids
31	33.62	0.06	1-Octadecanol	270.5	C_18_H_38_O	Alcohols
32	33.80	0.13	8,11-Octadecadienoic acid, methyl ester	294.5	C_19_H_34_O_2_	Fatty acids methyl esters
33	33.92	0.18	9-Octadecenoic acid, methyl ester, (E)-	296.5	C_19_H_36_O_2_	Fatty acids methyl esters
34	34.14	0.19	Phytol	296.5	C_20_H_40_O	Diterpenoids
35	34.40	0.03	Octadecanoic acid, methyl ester	298.5	C_19_H_38_O_2_	Fatty acids methyl esters
36	34.60	0.90	9,12-Octadecadienoic acid (Z,Z)-	280.4	C_18_H_32_O_2_	Fatty acyls
37	34.71	1.94	Oleic acid	282.5	C_18_H_34_O_2_	Fatty acids
38	34.991	0.81	Linoleic acid ethyl ester	308.5	C_20_H_36_O_2_	Fatty acids ethyl esters
39	35.10	1.55	Ethyl oleate	310.5	C_20_H_38_O_2_	Fatty acids esters
40	35.59	0.42	Octadecanoic acid, ethyl ester	312.5	C_20_H_40_O_2_	Fatty acids ethyl esters
41	35.69	0.32	Docosane	310.6	C_22_H_46_	Alkanes
42	37.38	0.14	Tricosane	324.6	C_23_H_48_	Acyclic alkanes
43	38.18	0.16	4,8,12,16-Tetramethylheptadecan-4-olide	324.5	C_21_H_40_O_2_	Terpenoids
44	38.92	0.11	Eicosanoic acid, ethyl ester	340.6	C_22_H_44_O_2_	Fatty acids ethyl esters
45	39.00	0.19	Tetracosane	338.7	C_24_H_50_	Alkanes
46	40.22	0.29	Desoxycorticosterone acetate	372.5	C_23_H_32_O_4_	Corticosteroids
47	40.42	0.09	Pregn-16-en-20-one, 3-(acetyloxy)-, (3.beta.,5.beta.)-	358.5	C_23_H_34_O_3_	Steroids
48	40.66	0.17	Hexadecanoic acid, 2-hydroxy-1-(hydroxymethyl)ethyl ester	330.5	C_19_H_38_O_4_	Fatty acids
49	41.22	0.10	1,2-Benzenedicarboxylic acid, bis(2-ethylhexyl) ester	390.6	C_24_H_38_O_4_	Phthalate esters
50	41.47	0.30	Oct-5-en-2-ol, 8-(1,4,4a,5,6,7,8,8a-octahydro-2, 5, 5, 8a-tetramethylnaphth-1-yl)-6-methyl-	332.6	C_23_H_40_O	Sesquiterpenoids
51	42.00	0.11	Docosanoic acid, ethyl ester	368.6	C_24_H_48_O_2_	Fatty acids ethyl esters
52	42.95	0.07	1,4-Pentadien-3-one, 1,5-diphenyl-	234.29	C_24_H_34_O_3_	*α*,β-unsaturated ketones
53	43.25	0.28	2-[4-Methyl-6-(2,6,6-trimethylcyclohex-1-enyl)hexa-1,3,5-trienyl]cyclohex-1-en-1-carboxaldehyde	324.5	C_23_H_32_O	Aromatic aldehydes
54	43.45	0.29	Lup-20(29)-en-3-ol, acetate, (3.beta.)-	468.8	C_32_H_52_O_2_	Triterpene esters
55	43.96	0.19	Pregn-4-ene-3,20-dione, 16,17-epoxy-, (16.alpha.)-	328.4	C_21_H_28_O_3_	Steroids
56	44.77	0.43	24-Norursa-3,12-diene	394.7	C_29_H_46_	Sesquiterpenes pentacyclic triterpene
57	45.12	0.14	3-Hydroxylanosta-8,24-dien-22-one	440.7	C_30_H_48_O_2_	Sesquiterpenes
58	45.60	0.88	Lanosta-8,24-diene-3,22-diol	442.7	C_30_H_50_O_2_	Triterpenoids
59	45.88	0.07	Alpha-tocospiro A	462.7	C_29_H_50_O_4_	Saponins
60	46.27	0.57	Nonacosane	408.8	C_29_H_60_	Alkanes
61	46.49	2.33	Dill apiole	222.27	C_12_H_14_O_4_	Phenylpropanoids
62	46.78	1.15	Hexacosanoic acid, methyl ester	410.7	C_27_H_54_O_2_	Fatty acid methyl esters
63	47.09	1.04	Lupeol	426.7	C_30_H_50_O	Triterpenoids
64	48.32	0.49	7-Dehydrodiosgenin	412.6	C_27_H_40_O_3_	Steroidal saponins
65	48.86	0.33	Tetratriacontane	478.9	C_34_H_70_	Alkanes
66	50.23	0.60	Campesterol	400.7	C_28_H_48_O	Sterols
67	50.64	0.61	Stigmasta-5,22-dien-3-ol, (3.beta.,22E)-	412.7	C_29_H_48_O	Phytosterols
68	51.34	5.93	Gamma.-sitosterol	414.7	C_29_H_50_O	Phytosterols
69	51.74	2.24	Lanosterol	426.7	C_30_H_50_O	Triterpenoids sterols
70	51.91	0.82	5.alpha.-Stigmast-7-en-3.beta.-ol,(24S)	414.7	C_29_H_50_O	Sterols
71	52.01	2.29	Lup-20(29)-en-3-one	424.7	C_30_H_48_O	Triterpenoids
72	52.18	1.61	Alpha-amyrin	426.7	C_30_H_50_O	Pentacyclic triterpenoids
73	52.39	6.39	9,19-Cycloergost-24(28)-en-3-ol, 4,14-dimethyl-, acetate, (3.beta.,4.alpha.,5.alpha.)-	468.8	C_32_H_52_O_2_	Steroids saponins
74	52.72	2.41	24-Methylenecycloartan-3-one	438.7	C_31_H_50_O	Triterpenoids
75	52.90	1.67	gamma. -Sitostenone	412.7	C_29_H_48_O	Phytosterols
76	53.305	0.99	23-(Phenylsulfanyl)lanosta-8,24-dien-3-ol	534.9	C_36_H_54_OS	Steroids
77	53.57	2.55	12-Oleanen-3-yl acetate,(3.alpha.)	468.8	C_32_H_52_O_2_	Triterpenoids
78	53.74	0.91	9,19-Cyclolanost-23-ene-3,25-diol, (3.beta.,23E)-	442.7	C_30_H_50_O_2_	Sterols
79	54.38	1.09	Betulinaldehyde	440.7	C_30_H_48_O_2_	Aldehydes
80	54.90	4.15	9,19-Cyclolanostan-3-ol, 24-methylene-, (3.beta.)-	440.7	C_31_H_52_O	Steroids
81	55.10	1.44	9,19-Cyclo-27-norlanostan-25-one, 3-(acetyloxy)-24-methyl-, (3.beta.,24R)-	484.8	C_32_H_52_O_3_	Steroids

**Table 4:** Antioxidant activities of EAHF.

Sample	ABTS	DPPH	FRAP	TAC	NOS
EAHF	159.5 ± 5	204.90 ± 4.90	258.36 ± 1.84	116.87 ± 0.62	93.36 ± 1.04

*Note:* All the results are expressed in mean ± standard deviation and calculated as mg of ascorbic acid equivalent per gram (A.A. eq/g) of dried extract.

**Table 5:** Enzyme inhibition % of EAHF.

Sample	Alpha-amylase inhibition %	Tyrosinase inhibition %	Urease enzyme inhibition%
EAHF	28.17	71.01	95.65217391
Standard	97.16	97.82	94.20289855

*Note:* Results are expressed as percentage inhibition.

**Table 6:** Thrombolytic and hemolytic activity of EAHF.

Sample	Thrombolytic activity (%)	Hemolytic activity (%)
EAHF	85.51	1.01
Standard	99.35	90.90

*Note:* Results are expressed as percentages.

**Table 7:** Lipinski's Rule of Five and solubility of best-docked compounds.

Phytocompounds	HBD	HBA	MWT	Lipophilicity	M.R	LR
Pregn-4-ene-3,20-dione, 16,17-epoxy-, (16.alpha.)-	0	3	328.45	3.07	93.02	0 vn
24-Norursa-3,12-diene	0	0	394.68	7.70	128.96	1 vn
Lanosta-8,24-diene-3,22-diol	2	2	442.72	5.89	138.20	1 vn
Lupeol	1	1	426.72	6.92	135.14	1 vn
7-Dehydrodiosgenin	1	3	412.60	4.83	121.12	1 vn
Lup-20(29)-en-3-one	0	1	424.70	6.82	134.18	1 vn
Alpha-amyrin	1	1	426.72	6.92	135.14	1 vn
23-(Phenylsulfanyl)lanosta-8,24-dien-3-ol	1	1	534.88	7.92	168.28	2 vn
12-Oleanen-3-yl acetate,(3.alpha.)	0	2	468.75	7.08	144.62	1 vn
9,19-Cyclolanost-23-ene-3,25-diol, (3.beta.,23E)-	2	2	442.72	6.00	136.34	1 vn

*Note:* MWT, molecular weight; vn, violation.

Abbreviations: HBA, hydrogen bond acceptor; HBD, hydrogen bond donor; MR, molar refractivity.

**Table 8:** Toxicity evaluation of EAHF.

Compound name	Predicted LD_50_ (mg/kg)	Predicted toxicity class	Hepatotoxicity	Carcinogenicity	Mutagenicity	Immunotoxicity	Cytotoxicity
Pregn-4-ene-3,20-dione, 16,17-epoxy-, (16.alpha.)-	6400	5	−	+	−	+	−
24-Norursa-3,12-diene	5000	5	−	−	−	+	−
Lanosta-8,24-diene-3,22-diol	2000	4	−	−	−	+	−
Lupeol	2000	4	−	−	−	+	−
7-Dehydrodiosgenin	8000	6	−	−	−	+	−
Lup-20(29)-en-3-one	5000	5	−	−	−		−
Alpha-amyrin	70,000	6	−	−	−	+	−
23-(Phenylsulfanyl)lanosta-8,24-dien-3-ol	1145	4	−	−	−	+	−
12-Oleanen-3-yl acetate,(3.alpha.)	3460	5	−	+	−	+	−
9,19-Cyclolanost-23-ene-3,25-diol, (3.beta.,23E)-	5000	5	−	−	−	+	−

*Note:* Positive, +; negative, −.

## Data Availability

The data that support the findings of this study are available from the corresponding author upon reasonable request.
