# Supplementary material for: Bioactivity Profiling of Chemically Characterized Extract of Saudi Jackfruit (Artocarpus heterophyllus) Using In Vitro and In Silico Approaches
Source: Scientifica (Cairo). 2025 Jun 22;2025:8015648. doi: 10.1155/sci5/8015648 (PMC12206570; doi:10.1155/sci5/8015648)
Supplement: Supporting Information — Additional supporting information can be found online in the Supporting Information section. [file 8015648.f1.docx]

**Supplementary materials**

**Bioactivity profiling of chemically characterized extract of Saudi Jackfruit (*Artocarpus heterophyllus*) using *in vitro* and *in silico* approaches**

**Table 1S:** EAHF docking against tyrosinase, alpha-amylase, and urease enzyme.

| **Peak no** | **Compound name** | **Tyrosinase**  **Binding**  **affinity** | **Alpha amylase**  **Binding**  **affinity** | **Urease**  **Binding**  **affinity** |
| --- | --- | --- | --- | --- |
| 1 | Hexanoic acid | -4.5 | -4.5 | -3.6 |
| 2 | 4H-Pyran-4-one, 2,3-dihydro-3,5-dihydroxy-6-methyl- | -5.6 | -5.9 | -4.3 |
| 3 | d-Mannose | -5.1 | -5.4 | -4.6 |
| 4 | Dodecane | -3.9 | -4.6 | -3.6 |
| 5 | 2-Decenal, (E)- | -4.6 | -4.6 | -3.9 |
| 6 | Tridecane, 3-methyl- | -5 | -4.7 | -4.2 |
| 7 | Tetradecane | -6.2 | -6.3 | -5.5 |
| 8 | 2(4H)-Benzofuranone, 5,6,7,7a-tetrahydro-4,4,7a-trimethyl-, (R)- | -6.6 | -6.3 | -5.3 |
| 9 | 1H-Cycloprop[e]azulen-7-ol, decahydro-1,1,7-trimethyl-4-methylene-, [1ar-(1a.alpha.,4a.alpha.,7.beta.,7a.beta.,7b.alpha.)]- | -6.9 | -6.8 | -6.3 |
| 10 | Hexadecane | -4.5 | -4.5 | -4.3 |
| 11 | 1,3,4,5-TETRAHYDROXYCYCLOHEXANECARBOXYLIC ACID | -5.3 | -6 | -4.6 |
| 12 | 9-Octadecen-1-ol, (Z)- | -4.8 | -4.8 | -4.3 |
| 13 | Pentadecane, 8-hexyl- | -4.6 | -4.9 | -4.4 |
| 14 | 6-Hydroxy-4,4,7a-trimethyl-5,6,7,7a-tetrahydrobenzofuran-2(4H)-one | -6.7 | -6.8 | -5.4 |
| 15 | Tetradecanoic acid | -4.5 | -4.7 | -4.4 |
| 16 | Heptadecane, 3-methyl- | -4.2 | -4.5 | -4.3 |
| 17 | Tetradecanoic acid, ethyl ester | -4.4 | -4.5 | -4.4 |
| 18 | Octadecane | -4.3 | -4.4 | -4 |
| 19 | Neophytadiene | -5.2 | -5.2 | -4.7 |
| 20 | 2-Pentadecanone, 6,10,14-trimethyl- | -5.5 | -5.2 | -4.6 |
| 21 | Phytol, acetate | -4.6 | -5.3 | -5 |
| 22 | Pentadecanoic acid, ethyl ester | -4.2 | -4.6 | -4.3 |
| 23 | 7,9-Di-tert-butyl-1-oxaspiro(4,5)deca-6,9-diene-2,8-dione | -6.5 | -7.4 | -6.8 |
| 24 | Z-(13,14-Epoxy)tetradec-11-en-1-ol acetate | -4.9 | -5 | -4.4 |
| 25 | Hexadecanoic acid, methyl ester | -4.1 | -5 | -4.2 |
| 26 | Octadecane, 2-methyl- | -4.2 | -4.8 | -4.4 |
| 27 | n-Hexadecanoic acid | -4.3 | -4.6 | -4.5 |
| 28 | Hexadecanoic acid, ethyl ester | -4.3 | -4.8 | -4.6 |
| 29 | Eicosane | -4.8 | -4.6 | -4.3 |
| 30 | Heptadecanoic acid | -4.6 | -5.1 | -4.8 |
| 31 | 1-Octadecanol | -5.1 | -4.9 | -4.3 |
| 32 | 8,11-Octadecadienoic acid, methyl ester | -4.3 | -5 | -4.4 |
| 33 | 9-Octadecenoic acid, methyl ester, (E)- | -4.7 | -4.8 | -4.6 |
| 34 | Phytol | -5 | -5.7 | -4.8 |
| 35 | Octadecanoic acid, methyl ester | -4.4 | -4.9 | -4.5 |
| 36 | 9,12-Octadecadienoic acid (Z,Z)- | -5.3 | -5.1 | -4.9 |
| 37 | Oleic Acid | -4.6 | -5.2 | -4.6 |
| 38 | Linoleic acid ethyl ester | -4.3 | -5.2 | -4.7 |
| 39 | Ethyl Oleate | -4.5 | -4.9 | -4.3 |
| 40 | Octadecanoic acid, ethyl ester | -4.6 | -4.8 | -4.3 |
| 41 | Docosane | -4.6 | -4.3 | -3.9 |
| 42 | Tricosane | -4.5 | -4.5 | -4 |
| 43 | 4,8,12,16-Tetramethylheptadecan-4-olide | -5.1 | -6 | -6 |
| 44 | Eicosanoic acid, ethyl ester | -4.6 | -4.7 | -4.4 |
| 45 | Tetracosane | -4.8 | -4.3 | -4.5 |
| 46 | Desoxycorticosterone Acetate | -7 | -8.3 | -7.5 |
| 47 | Pregn-16-en-20-one, 3-(acetyloxy)-, (3.beta.,5.beta.)- | -7.3 | -8.1 | -7 |
| 48 | Hexadecanoic acid, 2-hydroxy-1-(hydroxymethyl)ethyl ester | -4.7 | -4.9 | -4.6 |
| 49 | 1,2-Benzenedicarboxylic acid, bis(2-ethylhexyl) ester | -5.2 | -6.2 | -5.5 |
| 50 | Oct-5-en-2-ol, 8-(1,4,4a,5,6,7,8,8a-octahydro-2, 5, 5, 8a-tetramethylnaphth-1-yl)-6-methyl- | -7.1 | -6.9 | -6.4 |
| 51 | Docosanoic acid, ethyl ester | -4.5 | -4.9 | -4.3 |
| 52 | 1,4-Pentadien-3-one, 1,5-diphenyl- | -6.7 | -7.2 | -6.7 |
| 53 | 2-[4-methyl-6-(2,6,6-trimethylcyclohex-1-enyl)hexa-1,3,5-trienyl]cyclohex-1-en-1-carboxaldehyde | -7 | -7.8 | -6.8 |
| 54 | Lup-20(29)-en-3-ol, acetate, (3.beta.)- | -7.7 | -8.5 | -7.4 |
| 55 | Pregn-4-ene-3,20-dione, 16,17-epoxy-, (16.alpha.)- | -7.2 | -8.1 | -8.9 |
| 56 | 24-Norursa-3,12-diene | -8.4 | -9.1 | -8.5 |
| 57 | 3-Hydroxylanosta-8,24-dien-22-one | -7.8 | -8.7 | -7.1 |
| 58 | Lanosta-8,24-diene-3,22-diol | -8.1 | -8.6 | -7.2 |
| 59 | .alpha.-Tocospiro A | -5.8 | -6.9 | -6.5 |
| 60 | Nonacosane | -4.1 | -4.9 | -4.2 |
| 61 | Dill apiole | -6.4 | -6.2 | -5.4 |
| 62 | Hexacosanoic acid, methyl ester | -3.6 | -4.6 | -4.5 |
| 63 | lupeol | -8.2 | -9.5 | -7.4 |
| 64 | 7-Dehydrodiosgenin | -11.1 | -12.8 | -10.9 |
| 65 | Tetratriacontane | -4.2 | -4 | -4.1 |
| 66 | Campesterol | -7.9 | -8.3 | -7.1 |
| 67 | Stigmasta-5,22-dien-3-ol, (3.beta.,22E)- | -7.7 | -7.8 | -7.3 |
| 68 | .gamma.-Sitosterol | -7.5 | -8.2 | -6.6 |
| 69 | Lanosterol | -7.8 | -8.9 | -7.3 |
| 70 | 5.alpha.-stigmast-7-en-3.beta.-ol,(24S) | -7.8 | -8.2 | -7.1 |
| 71 | Lup-20(29)-en-3-one | -7.6 | -9.6 | -7.5 |
| 72 | .alpha.-Amyrin | -7.8 | -8.8 | -8.4 |
| 73 | 9,19-Cycloergost-24(28)-en-3-ol, 4,14-dimethyl-, acetate, (3.beta.,4.alpha.,5.alpha.)- | -7.4 | -8.3 | -7.1 |
| 74 | 24-Methylenecycloartan-3-one | -7.2 | -7.9 | -7 |
| 75 | .gamma.-Sitostenone | -6.9 | -8.2 | -6.8 |
| 76 | 23-(Phenylsulfanyl)lanosta-8,24-dien-3-ol | -9 | -8.4 | -7.2 |
| 77 | 12-Oleanen-3-yl acetate,(3.alpha.) | -7.8 | -8.5 | -7.9 |
| 78 | 9,19-Cyclolanost-23-ene-3,25-diol, (3.beta.,23E)- | -8.6 | -8.8 | -7.2 |
| 79 | Betulinaldehyde | -7.7 | -8.6 | -7.3 |
| 80 | 9,19-Cyclolanostan-3-ol, 24-methylene-, (3.beta.)- | -7.9 | -8.3 | -8.2 |
| 81 | 9,19-Cyclo-27-norlanostan-25-one, 3-(acetyloxy)-24-methyl-, (3.beta.,24R)- | -7.5 | -8.6 | -7.4 |
| 82 | Standard | -5.5^a^ | -7.9^b^ | -2.6^c^ |

a; kojic acid, b; Acarbose, c; Thiourea.
